# MicroRNAs and long non-coding RNAs during transcriptional regulation and latency of HIV and HTLV

**DOI:** 10.1186/s12977-024-00637-y

**Published:** 2024-02-29

**Authors:** Sergio P. Alpuche-Lazcano, Robert J. Scarborough, Anne Gatignol

**Affiliations:** 1 Virus-Cell Interactions Laboratory, Lady Davis Institute for Medical Research, 3999, Côte Ste Catherine St., Montréal, QC H3T 1E2 Canada; 2 Department of Medicine, Division of Experimental Medicine, McGill University, Montréal, QC H4A 3J1 Canada; 3 Department of Medicine, Division of Infectious Diseases, McGill University, Montréal, QC H4A 3J1 Canada; 4 Department of Microbiology and Immunology, McGill University, Montréal, QC H3A 2B4 Canada; 5 Present Address: National Research Council Canada, Montréal, QC H4P 2R2 Canada

**Keywords:** HIV, HTLV, microRNAs, Long non-coding RNAs, Transcription, Latency

## Abstract

Human immunodeficiency virus (HIV) and human T cell leukemia virus (HTLV) have replicative and latent stages of infection. The status of the viruses is dependent on the cells that harbour them and on different events that change the transcriptional and post-transcriptional events. Non-coding (nc)RNAs are key factors in the regulation of retrovirus replication cycles. Notably, micro (mi)RNAs and long non-coding (lnc)RNAs are important regulators that can induce switches between active transcription-replication and latency of retroviruses and have important impacts on their pathogenesis. Here, we review the functions of miRNAs and lncRNAs in the context of HIV and HTLV. We describe how specific miRNAs and lncRNAs are involved in the regulation of the viruses’ transcription, post-transcriptional regulation and latency. We further discuss treatment strategies using ncRNAs for HIV and HTLV long remission, reactivation or possible cure.

## Background

The cellular pathogenesis triggered by human retroviruses such as human immunodeficiency viruses (HIV) and human T cell leukemia viruses (HTLV) has been a subject of study for more than three decades with outstanding results, but the complexity of infection and latency is not fully elucidated [1, 2]. Retroviruses co-exist in cells in their proviral form within the cellular nucleus or in a replicative form producing virions. Therefore, they must undergo regulatory transcription by the host to switch from a transcriptionally silent form to an active one giving rise to latency or active replication. Among the cellular transcription regulating factors that retroviruses can encounter, long non-coding RNAs (lncRNAs) and microRNAs (miRNAs) play key roles in virus modulation.

lncRNAs are defined as non-coding (nc) transcripts longer than 200 nucleotides (nt) [3, 4]. lncRNAs regulate various nuclear and cytoplasmic mechanisms of gene expression such as chromatin architecture [5, 6], assembly and function of different nuclear bodies [7–9], and transcriptional and post-transcriptional events [4, 10, 11]. miRNAs are ncRNAs that typically regulate gene expression through the RNA interference (RNAi) pathway by post-transcriptional mechanisms that lead to the repression or degradation of a target mRNA [12–16].

As RNA molecules, lncRNAs and miRNAs have different characteristics and regulatory mechanisms within cells and there can be crosstalk between them. lncRNAs that contain complementary sites for miRNAs can act as sponges by binding miRNAs and inhibiting their post-transcriptional gene control activity [17, 18]. Conversely, miRNAs can interact with lncRNA, thereby controlling their functions or triggering their decay [19, 20]. Furthermore, miRNAs and lncRNAs can compete for the same mRNA target and some miRNAs can be derived from lncRNAs [21]. The expression, interplay and functionality of these types of ncRNAs can be modified, derived from or used by retroviruses.

The evidence of the interplay between lncRNAs, miRNAs and retroviruses has been studied in major human retroviruses [22]. In this review, we will cover the association, interplay and activity of lncRNAs and miRNAs induced, derived or regulated mainly by HIV and HTLV during transcription, post-transcriptional regulation and latency. Because most studies have been carried out on HIV-1 and HTLV-I, this review will refer to these viruses as HIV and HTLV unless otherwise indicated.

## lncRNA and miRNA biogenesis and function

Most lncRNAs are derived from precursor mRNAs and are transcribed and processed similarly to mRNAs. Although many of them retain a 5′ cap and 3′ poly(A) tail, not all lncRNAs share these features or origins as others have distinct biogenesis pathways [23]. For instance, long intergenic non-coding RNAs (lincRNAs) are generated by a phosphorylated-dysregulated RNA polymerase (Pol) II [24]. Despite lncRNAs sharing similar characteristics with mRNAs, they contain fewer exons, weaker internal splicing signals and alternative polyadenylation. They are often inefficiently spliced and retained in the nucleus [25–27]. lncRNAs rich in A/U with one or very few exons can be exported to the cytoplasm through nuclear export factor 1 (NXF1) and the transcription export (TREX) complex [28]. In the nucleus, lncRNAs have a diverse array of functions such as chromatin regulation of architecture and gene expression [5, 6]. It is likely that negative charges of lncRNAs and positively charged histone tails lead to relaxing chromatin favoring DNA regulation by lncRNAs [29]. The lncRNA-chromatin interaction can be facilitated by proteins [30, 31] or carried out by direct interaction with DNA which can form triple helices or R-loops [32, 33]. lncRNAs can regulate gene expression at a transcriptional level at neighboring and non-neighboring loci by hindering transcription factors or RNA Pol II [34], regulating the formation of genomic domains [35], assembling nuclear scaffolds and condensates such as paraspeckles [36], speckles [7] and perinuclear compartments [37]. At the post-transcriptional level, lncRNAs can modulate gene expression by hijacking proteins involved in mRNA turnover or cell signaling and by sponging miRNAs or other regulatory RNAs [38–41].

In the RNAi pathway there are three types of short double-stranded (ds)RNAs of ~ 23 nt commonly recognized as functional RNAi molecules: small interfering (siRNAs), short-hairpin RNAs (shRNAs) and miRNAs. While the first two molecules are either produced in plants or synthesized artificially, miRNAs are the primary small ncRNAs used naturally by the RNAi pathway in mammalian cells. A distinguishing characteristic of miRNAs compared to siRNAs and shRNAs is that they are not completely complementary to their corresponding target mRNA and because of that, they do not direct the RNAi pathway to cleave the target mRNA, but instead lead to translational repression or targeted degradation. Most miRNAs are synthesized by a canonical pathway, which starts with the synthesis of a primary (pri)-miRNA in the nucleus by RNA Pol II [42]. pri-miRNAs are cleaved by the nuclear enzyme Drosha in complex with DiGeorge syndrome critical region 8 (DGCR8) that produce precursor (pre)-miRNAs which are exported to the cytoplasm by Exportin-5 [43, 44]. In the cytoplasm, pre-miRNAs are processed by Dicer in complex with its co-factors, the TAR RNA binding protein (TRBP), or the protein kinase R activator (PACT) to produce double-stranded miRNAs [45–48]. After being loaded onto an Argonaute (Ago) protein, one of the strands will be removed and the remaining single-stranded guide RNA will hybridize to its target sequence, which will trigger the mRNA silencing process by translational repression and degradation in processing (P) bodies [49]. miRNAs can also be generated by non-canonical pathways in which Drosha and DGCR8 are not required because these miRNAs are not directly transcribed from RNA Pol II but derived from other types of nuclear RNAs [50, 51].

miRNA guide strands generally do not exhibit complete complementarity to their target sequences located mostly in the 3′ untranslated region (UTR) of mRNAs. The miRNA seed region, located 2 to 8 nt from the 5′ end, along with nucleotides at position 13–16 are sufficient to repress mRNAs [52]. miRNAs possess multiple potential mRNA targets, and conversely, many mRNAs can be targeted by different miRNAs [53]. Because of these characteristics, it is difficult to accurately estimate the entire miRNA targetome. Different algorithms use the canonical rule, but this is a major challenge when bulges, G:U wobbles and “seedless” interactions are considered [54]. Nevertheless, according to the latest estimates based on protein-coding genes, miRNAs could regulate up to 60% of the total proteome [55]. Overall, lncRNAs and miRNAs are master regulators in a large number of different cell mechanisms and pathways and are of high importance during productive replication and latency of HIV and HTLV.

## HIV and HTLV transcription in replication and latency

Since the identification of HIV and HTLV in the early 1980s, both viruses have been comprehensively studied. To understand the crosstalk between lncRNAs, miRNAs and HIV or HTLV, here, we will briefly recapitulate both virus transcriptional and post-transcriptional events during replication and latency.

Within the HIV provirus long terminal repeats (LTR), the unique region 3 (U3) harbours the promoter and regulatory binding sites that allow the recruitment of RNA Pol II and the production of transcripts at low levels [56–58]. The repeated (R) unique region 5 (U5) encodes the trans-activation response (TAR) element, present at the 5′ end of each HIV RNA, followed by the full-length mRNA. In the early phase of the replication cycle, transcripts are completely spliced, exported to the cytoplasm and translated into Tat, Rev, and Nef proteins [59]. After translation, Tat is imported into the nucleus and it recruits the positive transcription elongation factor b (P-TEFb), composed of cyclin-dependent kinase (CDK) 9 and cyclin T1 (CycT1) bound to the 7SK complex [60]. 7SK is an abundant lncRNA of 332 nt localized mainly in the nucleus [61]. Host proteins such as La-related protein 7 (LaRP7) and methylphosphate capping enzyme (MePCE) contribute to the 7SK RNA structure and stability whereas, hexamethylene bisacetamide-induced proteins (HEXIM)1/2 bind to P-TEFb and regulate its release from the 7SK RNA [62]. Tat recruits P-TEFb and binds to the bulge of TAR RNA, whereas CycT1 binds to the loop. As a result of this interaction, P-TEFb becomes activated and hyperphosphorylates RNA Pol II. P-TEFb then associates with the super elongation complex (SEC), a scaffold formed by AF4/FMR2 Family Members 1 & 4 (AFF1/4), eleven nineteen leukaemia (ENL)/ALL1-fused gene from chromosome 9 protein (AF9) and eleven nineteen lysine-rich leukaemia protein (ELL2) that facilitates HIV transcriptional elongation [63, 64]. These modifications and complex formation enhance the processivity of RNA Pol II and lead to a significant increase in viral transcripts, often reaching several 100-fold amplification [61, 65, 66]. Unspliced and partially spliced viral RNAs are restricted from nuclear export. To overcome this, nuclear imported HIV Rev binds the Rev response element (RRE), a highly structured, short cis-acting RNA element contained in the Env region [67, 68]. By binding to chromosomal maintenance 1 (CRM1), DEAD-box helicase 3 (DDX3), host up-frameshift protein 1 (UPF1), and Ran (GTP), the Rev-RRE complex enables the transcript to reach the nuclear pore and exit the nucleus [69, 70]. In the cytoplasm, Ran(GTP) is dephosphorylated inducing the disassembly of the Rev and RRE, which allows the translation of Gag/Gag-Pol and its packaging with the genomic RNA [69, 71].

The integrated HTLV genome harbors its promoter within the LTR U3 region and starts transcription in the R region followed by U5 translation. Its genome contains a unique region called pX, located between the env gene and the 3′LTR. This region overlaps in open reading frames (ORFs) III and IV that code for Tax and Rex proteins which are generated from completely spliced viral mRNA as part of the early translation events similar to Tat and Rev in HIV [72]. Tax and Rex proteins contain nuclear localization signals (NLS) that allow them to enter the nucleus and participate in the viral transactivation and long transcript export processes. The HTLV U3 region contains the Tax responsive element I (TRE-1), a three discontinued (A, B, C) 21 base pair (bp) repeats. The B domain encompasses a 5–8 bp viral cAMP response element (vCRE) which is the site that binds the cAMP response element binding (CREB) protein. Tax binds to CREB and to CREB-binding protein (CBP)/p300 and forms a complex including coactivators p300/CBP-associated factor (P/CAF) and transducer of regulated CREB protein (TORC) that initiates transcription [73–76]. The recruitment of CBP/p300 to the Tax/CREB complex hyperacetylates histones that strongly favor chromatin remodeling [77]. This action causes the displacement of nucleosomes from the promoter region, leading to increased transcription at the HTLV promoter [73]. Meanwhile, Rex proteins accumulate in the nucleus allowing partially spliced (env) and unspliced (gag-pro-pol) mRNAs to be exported to the cytoplasm. Rex recruits CRM1 and binds to the Rex-responsive element (RexRE) present in unspliced and partially spliced transcripts, which allows their exit from the nucleus to the cytoplasm for structural protein translation and genomic RNA packaging [78]. HTLV also initiates transcription from the minus-strand 3′ end of the proviral DNA generating HTLV basic leucine zipper factor (HBZ), a transcriptional suppressor of Tax and a main player in maintaining infected T cells and determining their immunophenotype [79].

HIV and HTLV can undergo latency when viruses accumulate and persist in a non-productive state of infection in specific cell types and anatomical sites (viral reservoirs), remaining transcriptionally silent, but retaining the capacity to produce infectious virus particles upon stimulation [80, 81]. During acute infection by HIV, most CD4^+^ T effector cells die but a small subset enter into G_0_ phase (resting memory cells), a quiescent state that is favorable for latency [82–84]. These latently infected cells are extremely rare (1/1 × 10^6^ resting CD4^+^ T cells) with a half-life of 44 months and it has been estimated that it would require 73.4 years to eradicate these cells under antiretroviral therapy (ART) [85]. While CD4^+^ T cells constitute the main reservoir, myeloid cells like monocytes and macrophages may also serve as HIV latent reservoirs [86].

In cells latently infected with HIV, the provirus could be located in the same orientation as actively transcribed genes possibly inducing HIV transcriptional interference or in non-genic regions promoting deep latency defined as impossible to difficult-to-reactivate proviruses using latency reversing agents (LRAs) [87, 88]. For intact HIV proviruses, the deepness of viral latency is multifactorial, but is increased by the distance from accessible chromatin and active transcriptional units as well as positions with opposite orientation to host genes [88]. Furthermore, increased silencing through the promoter of the latent HIV provirus occurs by epigenetic regulation including histone deacetylation and DNA methylation by methyl-CpG binding domain protein 2 (MBD2) [89, 90]. In addition, ubiquitin-like with PHD and RING finger domain 1 (UHRF1) factor binds to the HIV latent promoter and is an epigenetic repressor [91]. Methylation of CpG islands on the DNA of the Env region also occurs in intact noninduced proviral sequences, suggesting a role of these sequences in latency [92]. Resting CD4^+^ T cells retain HIV transcription initiation factors such as nuclear factor kappa B (NF-κB) and nuclear factor of activated T cells (NFAT) in the cytoplasm whereas CycT1 and HIV transcripts are regulated by a subset of miRNAs [82, 83, 93]. The presence of these factors explains why cytokines and T cell receptor (TCR) activation can reverse these processes and activate HIV transcription [94].

HTLV-infected CD4^+^ T cells undergo latency using mechanisms that are similar to HIV’s but also some specific ones. HTLV provirus is integrated in the cellular DNA while viral RNA is mainly transmitted by cell-to-cell contacts and rarely present in the plasma of infected subjects regardless of the status of the disease [95]. Protein phosphatase 2A (PP2A) is a major factor for HTLV DNA integration which might occur in the same-sense genome orientation referred to as transcriptional interference [81]. Importantly, the interplay between Tax and HBZ plays a major role in latency. Tax protein is highly expressed, immunogenic and therefore downregulated by the immune system. In contrast, HBZ is poorly immunogenic and constantly expressed at low levels. HBZ downregulates Tax by a dual mechanism: it competes directly with Tax binding to CBP/p300 and hinders the interaction of ATF/CREB and CBP/p300 with the LTR [79]. It is also likely that epigenetic regulation such as cytosine methylation of the 5’LTR further contributes to the dormant state of HTLV [96]. Additionally, for both HIV and HTLV replication and latency, lncRNAs and miRNAs play important roles as key regulators of transcriptional and post-transcriptional events during replicative or latency states.

## lncRNAs during transcriptional regulation and latency of HIV

**Fig. 1:**
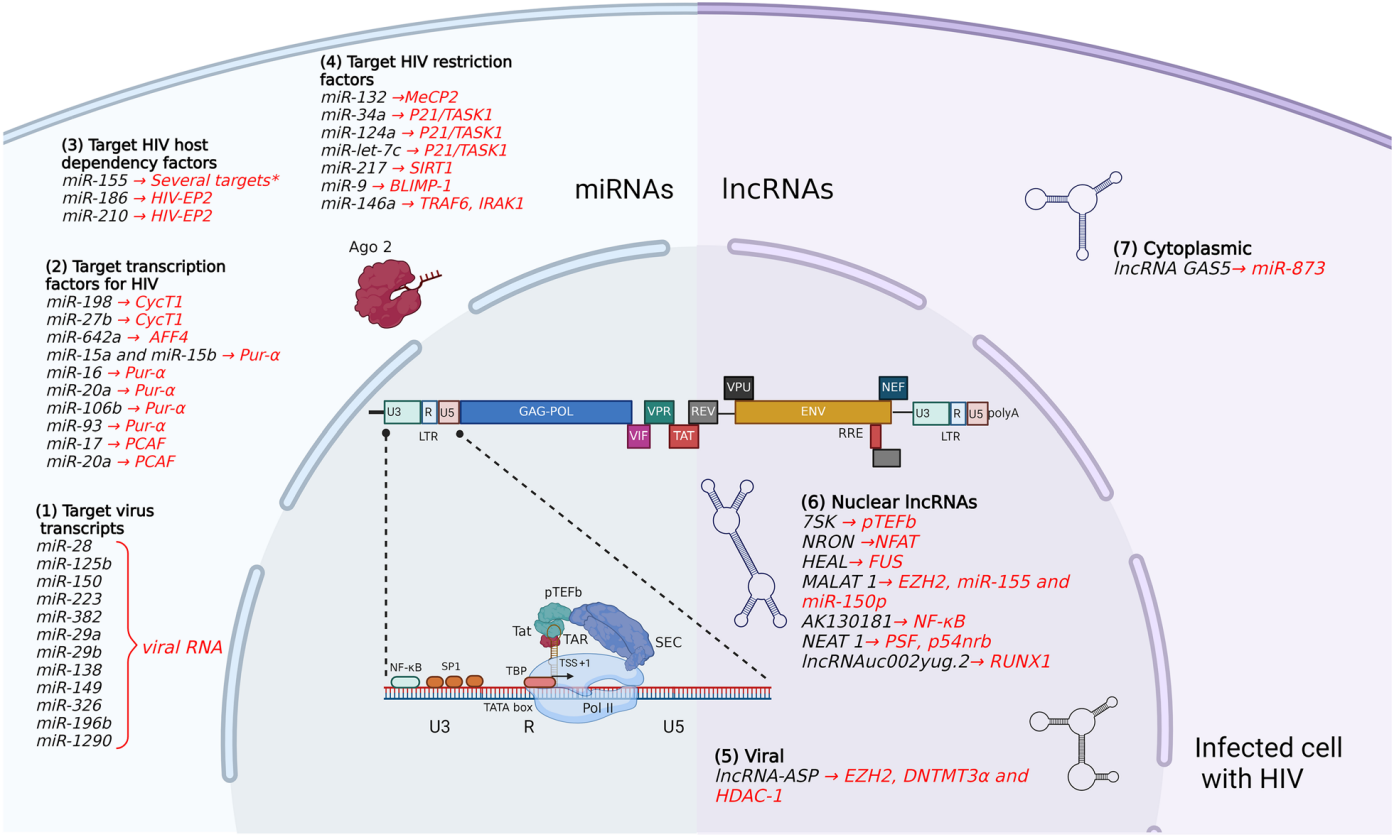
Dysregulated miRNAs and lncRNAs in HIV-infected cells. A schematic diagram of the HIV genome within an infected cell highlighting the transcription processes along with the dysregulation of miRNAs and lncRNAs triggered by the virus infection. During the late phase of HIV infection, Tat translocates to the cell nucleus, interacts with pTEFb and brings Tat-pTEFb complex to the TAR element. This complex allows pTEFb to hyperphosphorylate RNA Pol II initiating the virus transactivation process. The different miRNAs and lncRNA listed are dysregulated, modified or have a reported function during HIV transcription and latency. (left) Cellular miRNAs that target 1) viral transcripts, 2) transcription factors, 3) HIV host dependency factors, 4) HIV restriction factors. (right) LncRNAs that are 5) derived from ASP HIV transcript, 6) nuclear lncRNA and 7) cytoplasmic lncRNAs. *Refer to Table 1. Figure created with Biorender

lncRNAs involved in HIV replication and latency have different origins and act at different stages (Fig. 1; Table 1). Remarkably, HIV might transcribe its own lncRNAs. Indeed, the antisense mRNAs are transcribed from a TATA-less promoter in the 3′ LTR region [97–100]. When exported to the cytoplasm, these mRNAs code for the HIV antisense protein (ASP), which induces autophagy [101–103]. Several alternative forms of the antisense RNA have been described ranging from 2.2 to 2.6 kb in length due to the transcription from the Tat-independent, TATA-less promoter with multiple initiation sites [102, 104, 105]. They are mainly localized in the nucleus and nuclear ASP transcripts are considered as lncRNAs [106]. They can recruit the enhancer of Zeste homolog 2 (EZH2), DNA methyl transferase 3 alpha (DNTMT3α), and histone deacetylase 1 (HDAC-1), which are chromatin remodeling factors. These lncRNAs accumulate at the 5′ LTR and function in epigenetic control by modulating transcription and possibly contributing to viral latency [107].

**Table 1:** Target and effects of cellular lncRNAs and miRNAs during HIV transcriptional and post transcriptional events

lncRNA	Target	Outcome	References
lncRNA ASP (viral)	EZH2, DNTMT3α and	Epigenetic control	[107]
	HDAC-1		
RNA 7SK	pTEFb	Stabilizes pTEFb complex	[111]
NRON	NFAT	Decreases HIV transcription	[112]
HEAL	FUS	Increases HIV transcription by epigenetic regulation	[114]
MALAT-1	EZH2, miR-155,150-5p	Increases HIV transcription, favours HIV reactivation by epigenetic regulation	[115]
			[116]
AK130181	NF-κB	Decreases HIV transcription	[117]
NEAT-1	PSF, p54nrb	Paraspeckle formation and	[118]
	(Paraspeckle proteins)	HIV transcripts retention in nucleus	
uc002yug.2	RUNX1	Increases HIV transcription	[121]
		by inhibiting a repressor and increasing Tat	
GAS5	miR-873	Decreases HIV replication	[122]

miRNA	Target	Outcome	References
miR-28, miR-125b,	RNA viral genome	Downregulation of HIV expression	[136, 138–141, 233]
miR-150, miR-223, miR-382, miR-29a, miR-29b, miR-138, miR-149, miR-326, miR-196b, miR-1290			
miR-198, miR-27b	CycT1	Downregulation of HIV transcription	[93, 142]
miR-642a	AFF4	Downregulation of HIV transcription	[143]
miR-15a, miR-15b,			
miR-16, miR-20a,	Pur-α	Downregulation of HIV transcription	[145]
miR-106b, miR-93			
miR-17, miR-20a	PCAF	Downregulation of HIV transcription	[146]
miR-155	ADAM10, TNPO3, TRIM32,	Downregulation of HIV transcription	[147, 148]
	NUP153, LEDGF/p75		
miR-186, miR-210	HIV-EP2	Reduction of viral replication	[149]
miR-132	MeCP2	Upregulation of HIV transcription	[150]
miR-34a, miR-124a,	P21/TASK1	Upregulation of HIV transcription	[153]
miR-let-7c			
miR-217, miR-34a	SIRT1	Upregulation of HIV transcription	[155, 156]
miR-9	BLIMP-1	Upregulation of HIV transcription	[158]
miR-146a	TRAF6, IRAK1	Upregulation of HIV replication	[161]

Various host lncRNAs contribute to HIV replication or latency by acting on viral transcription [108–110]. As previously stated, lncRNA 7SK scaffolds a large group of proteins including pTEFb [60]. Besides Tat, cellular factors can contribute to pTEFb recruitment to the 5′LTR. 7SK naturally undergoes pseudouridine-acidification at U250 by DCK1-BoxH/ACA ribonucleoprotein complex which stabilizes the 7SK structure and retains pTEFb. The lack of pseudouridylation leads to 7SK degradation, thereby promoting the release of pTEFb and enhancing viral replication [111]. Another lncRNA is the non-coding repressor of NFAT (NRON) that regulates HIV transcription depending on the stage of the replication cycle [112]. Cytoplasmic NRON forms a ribonucleoprotein complex with the transcription factor NFAT inhibiting its translocation to the nucleus and preventing its enhancer activity on the HIV promoter. During the early phase of virus replication, the accessory protein Nef reduces NRON levels which allows NFAT to be imported to the cell nucleus. Conversely, during the late phase of the replication cycle, HIV produces Vpu, enhancing NRON expression and hence inhibiting the nuclear import of NFAT [112]. In addition, NRON expression degrades Tat, which likely also contributes to HIV latency [113]. HIV-enhanced lncRNA (HEAL) is a lncRNA that is upregulated in monocyte-derived macrophages (MDMs) and lymphocytes upon infection with HIV and is an enhancer of viral replication [114]. HEAL forms a complex with the RNA binding protein FUS, which positively regulates HIV transcription by binding to the HIV promoter and recruiting the histone acetyltransferase p300. The HEAL-FUS complex also binds to the promoter of the *CDK2* gene, promoting CDK2 expression, which phosphorylates CDK9 within pTEFb and further enhances transcriptional elongation [114]. The metastasis-associated lung adenocarcinoma transcript 1 (MALAT-1) is another lncRNA involved in promoting transcription of HIV by relieving epigenetic silencing. Indeed, HIV infection induces the expression of MALAT-1, which removes EZH2 from the polycomb repressive complex 2 (PRC2), thereby preventing EZH2 recruitment to the LTR promoter. Therefore, in the presence of MALAT1, PRC2 can no longer silence HIV transcription by histone methylation [115]. MALAT1 also acts as a sponge to prevent miR-155 and miR-150-5p inhibitory activity of suppressor of cytokine signaling 1 (SOCS1), promoting HIV replication. Therefore, low levels of MALAT1 may play a role in latency and its induction may promote HIV reactivation [116]. Additionally, lncRNA AK130181 is highly expressed in latently infected CD4^+^ T cells and can suppress HIV promoter activity in an NF-κB-dependent manner [117].

In addition to the transcriptional activity on the HIV promoter, some lncRNAs can act at a post-transcriptional level. Nuclear enriched abundant transcript 1 (NEAT1) is a lncRNA necessary for paraspeckle formation in the nucleus [118]. During the late phase of HIV replication, NEAT1 stores unspliced HIV transcripts in nuclear paraspeckles, decreasing overall virus replication. Upon NEAT1 downregulation, Rev has access to unspliced HIV transcripts allowing their nuclear export, translation and RNA packaging into new virions [118]. lncRNA uc002yug.2 derived from LINC1426, is a lncRNA that promotes alternative splicing of runt-related transcription factor 1 (RUNX1), a repressor that acts by binding to the HIV promoter [119, 120]. The induction of RUNX1 isoforms by lncRNA uc002yug.2 activates HIV transcription in latently infected cells, which further upregulates Tat expression [121]. Conversely, the lncRNA growth arrest-specific transcript 5 (GAS5) can sponge miR-873, a miRNA that promotes HIV replication [122]. Furthermore, the up-regulation of lncRNAs plasmacytoma variant translocation 1 (PVT1) and RP11-347C18.3 in HIV-infected memory CD4^+^ T cells and their association with the spliceosome pathway suggest a role in latency establishment and maintenance [123].

## miRNAs during transcriptional regulation and latency of HIV

Many host miRNAs have been found dysregulated differently depending on the cell type and HIV stage of infection or latency. This complexity is notably heightened by miRNAs that are possibly encoded by the virus genome.

### Viral miRNAs

The production of viral (v)miRNAs by HIV remains a subject of controversy because of their low abundance and the difficulty to detect them. Bioinformatic prediction analyses have revealed that various regions within the HIV genome exhibit potential sites for vmiRNA synthesis. These regions in viral transcripts encoded from the 5′ and 3′LTR, Gag-CA, Pol and Nef present stem-loops similar to pri-miRNAs or pre-miRNAs [124, 125]. For instance, vmiRNA miR-H1, produced from the 3′ LTR, targets the apoptosis antagonizing transcription factor (AATF) mRNA and consequently Bcl-2, c-myc, Par-4 and Dicer. It also downregulates the cellular miR-149 that targets Vpr, although sequence variability of miR-H1 suggests a non-uniform function [126, 127]. vmiRNA miR-N367 generated from the Nef coding sequence in HIV-infected MT-4T cells, decreases viral expression [128]. However, another study carried out on HIV-infected lymphocytic SupT1 cells and analysing HIV miRNAs by deep sequencing did not retrieve miR-N367 [125]. Computational prediction and deep-sequencing also revealed miR-H3 located within the HIV RT region. Overexpressed miR-H3, enhances HIV transcription by interacting with the TATA box in the promoter [129]. Interestingly, the TAR RNA at the 5′ end of each HIV RNA resembles a pre-miRNA which can be processed by Dicer into TAR-miR-5p and 3p. They can be incorporated into Ago2 complexes and could regulate apoptotic genes to keep a balance between cell apoptosis and survival [125, 130–132]. Despite these discoveries, several other studies using deep sequencing assays in either cell lines or primary HIV-infected cells failed to detect any small RNA derived from the virus or showed that less than one percent of small RNAs were vmiRNAs [133–135]. Due to the low abundance of HIV-encoded vmiRNAs, their functional relevance in infected lymphocytes is still to be demonstrated. Furthermore, host miRNAs are present in the cell and they are regulated during HIV replication and latency. They can target the viral RNA, transcription factors, host dependency factors (HDF) or host restriction factors (HRF).

### Host miRNAs targeting HIV RNA

Resting CD4^+^ T cells that carry an integrated HIV genome do not produce virus and are in a latent state. The analysis of their miRNA expression shows a correlation with a subset of highly produced miRNAs including miR-28, miR-125b, miR-150, miR-223 and miR-382 that directly interact with the viral RNA and contribute to the inhibition of virus replication [136]. Similarly, this effect is observed in monocytes which also express miR-28, miR-150, miR-223 and miR-382 [137]. A decrease of these miRNAs in monocytes increases HIV replication, whereas their expression in macrophages reduces HIV replication [137]. miR-29a and miR-29b have a strong ability to reduce viral replication (especially miR-29a) by targeting a sequence in the Nef coding region and consequently repressing all HIV transcripts, making them potential pro-latency agents [138, 139]. miR-149 targeting Gag RNA, miR-138 targeting Env RNA, and miR-29b, miR326 targeting Nef-U3 regions remained active and decreased HIV replication 6 days post-infection suggesting a long-term effect in latency [140]. miR-196b and miR-1290 targeting Nef/U3 sequences, identified as upregulated miRNAs in latently infected cells repressed HIV production and modulated viral infectivity, suggesting a possible involvement in viral latency [141]. Therefore, to date, twelve cellular miRNAs that target different regions of HIV have been identified and characterized for their contribution to decrease viral RNA, reduce viral replication and possibly contribute to HIV latency by directly limiting RNA expression (Fig. 1; Table 1).

### Host miRNAs targeting transcription factors

Several miRNAs target transcription factors and affect HIV transcription in a cell type dependent manner. During the differentiation process of monocytes into macrophages, CycT1 expression levels are initially low. This characteristic is associated with the production of miR-198, which plays a role in regulating CycT1. As a result, miR-198 is able to suppress HIV transcriptional elongation and consequently viral replication [142]. Likewise, resting CD4^+^ T cells produce miR-27b, which targets and reduces the expression of CycT1, thereby inhibiting viral replication. However, when CD4^+^ T cells are activated, miR-27b is downregulated, creating a favorable environment for viral replication [93]. In addition, miR-29b, miR-150 and miR-223 are also downregulated upon CD4^+^ T cell activation and indirectly decrease CycT1 expression [93]. Our group has recently identified miR-642a-3p that hinders HIV transcriptional elongation by targeting AFF4 mRNA coding for a protein of the SEC complex. During HIV replication, miR-642a-3p is retained on a complex formed by Dicer and Gag, which prevents its downregulating activity on AFF4 [143]. The Pur-α protein binds HIV TAR/Tat, thereby increasing viral trans-activation. Endogenously, monocytes produce miR-15a, miR-15b, miR-16, miR-20a, miR-106b, and miR-93 that repress Pur-α leading to a major decrease of HIV transcription [144, 145]. The histone acetyltransferase and Tat cofactor, P/CAF, can also be targeted by the cluster miR-17-5p and miR-20a leading to a decrease of HIV transcription [146].

### Host miRNAs targeting HIV HDFs

In addition to transcription factors, HDFs are required for HIV replication. Several host miRNAs can counteract the expression of HIV HDFs. Upon activation of either Toll-like receptor (TLR) 3 or TLR4, MDMs increase the expression of miR-155. This miRNA significantly reduces HIV replication by targeting a disintegrin and metalloproteinase (ADAM)10, transportin-3 (TNPO3), nucleoporin NUP153, and the transcriptional co-activator lens epithelium-derived growth factor (LEDGF)/p75, which are fundamental factors during trafficking and nuclear import of the viral pre-integration complex [147]. In addition, experiments in the latently infected T cell line, J-Lat 5A8, demonstrated that miR-155 also targets the tripartite motif containing 32 (TRIM32), an enhancer of NF-κB and hence, prevents HIV transcription and favours HIV latency [148]. Another example of dual activity over one gene are miR-186 and miR-210 which are upregulated during HIV infection and repress the expression of the HIV-enhancer binding protein 2 (EP2). A decrease of HIV-EP2 leads to reduced HIV gene expression and may contribute to HIV latency [149].

### Host miRNAs targeting HIV HRF

HRFs prevent HIV replication by counteracting viral factors at different steps in the viral replication cycle. Several miRNAs can counteract HRFs and enhance HIV replication. Activated CD4^+^ T cells show an enhanced expression of miR-132. The overexpression of miR-132 in Jurkat cells leads to the posttranscriptional repression of Methyl-CpG binding protein 2 (MeCP2) gene, an inhibitory factor of HIV integration that antagonizes LEDGF [150]. p21 and TWIK-related acid-sensitive K (TASK)1 are also HRFs that reduce a deoxyribonucleotide triphosphate (dNTPs) pool necessary for virus replication and inhibit HIV Vpu, respectively [151, 152]. Following HIV infection of HeLa cells expressing C–C chemokine receptor (CCR)5, let-7c, miR-34a and miR-124a are upregulated and inhibiting them reduces HIV replication. let-7c reduces the level of p21 mRNA while miR-34a and miR-124a target TASK1, thereby contributing to HIV replication [153]. Sirtuin 1 (SIRT1) is a deacetylase that acts on histones and transcription factors. Upon HIV infection, SIRT1 deacetylates HIV Tat. In turn, Tat inhibits SIRT1 HDAC domain, which blocks its ability to deacetylate NF-κB p65 subunit, leading to hyperactivation of HIV-infected cells [154]. Tat induces the production of miR-34a and miR-217 which target SIRT1, decrease its expression and Tat-induced acetylation of NF-κB, leading to increased transcription by NF-κB and enhanced trans-activation by Tat [155, 156]. B lymphocyte-induced maturation protein-1 (BLIMP-1) is an HIV transcriptional restriction factor which is overexpressed in CD4^+^ memory T lymphocytes. BLIMP-1 acts by direct binding to the 5′LTR downstream of the + 1 transcription start site and limits basal HIV transcription, which contributes to HIV latency. BLIMP-1 also represses interleukin (IL)-2 transcription. Experiments in lymphoma HUT78 cell lines demonstrated that miR-9-5p downregulates BLIMP-1, which consequently increases HIV and IL2 transcription [157, 158]. Of note, peripheral blood mononuclear cells (PBMCs) of HIV elite controllers (EC) showed low concentrations of miR-9-5p suggesting the importance of this miRNA in HIV transcription [159]. Another observation in a CD4^+^ T cell line showed an upregulation of miR-34c-5p in activated CD4^+^ T cells associated to increased HIV replication [160]. The results of this study were unexpected because overexpression of miR-34c-5p results in a decrease of P/CAF, a transcriptional enhancer of HIV, which in theory should be otherwise. The authors suggested that miR-34c-5p may affect a high array of genes, which overall increase HIV replication. miR-146a also affects a large number of genes by direct inhibition of tumor necrosis factor (TNF) receptor-associated factor 6 (TRAF6) and IL-1 receptor-associated kinase 1 (IRAK1) and consequently represses the innate immune response and increases viral replication [161]. Indeed, engineered lymphocytic MT2 cells abrogated for miR-146a expression and infected with HIV showed an upregulation of cytokines and HIV restriction factors such as myxovirus resistance protein B (MxB), interferon (IFN) induced transmembrane protein 1 (IFTIM1) and tetherin, all restricting HIV replication.

Overall, the crosstalk between miRNAs and HIV is highly complex and leads to the up and downregulation of a large array of genes. The large number of studies that have explored relationships between ncRNAs and HIV have generated new knowledge that greatly enhance our understanding of HIV biology and could also help to find novel strategies to either block or re-activate latent virus in reservoirs to potentially cure people infected with HIV.

## ncRNAs and miRNAs during HTLV replication and latency

### lncRNAs as transcriptional enhancers promoting cell proliferation in HTLV-infected cells

**Fig. 2:**
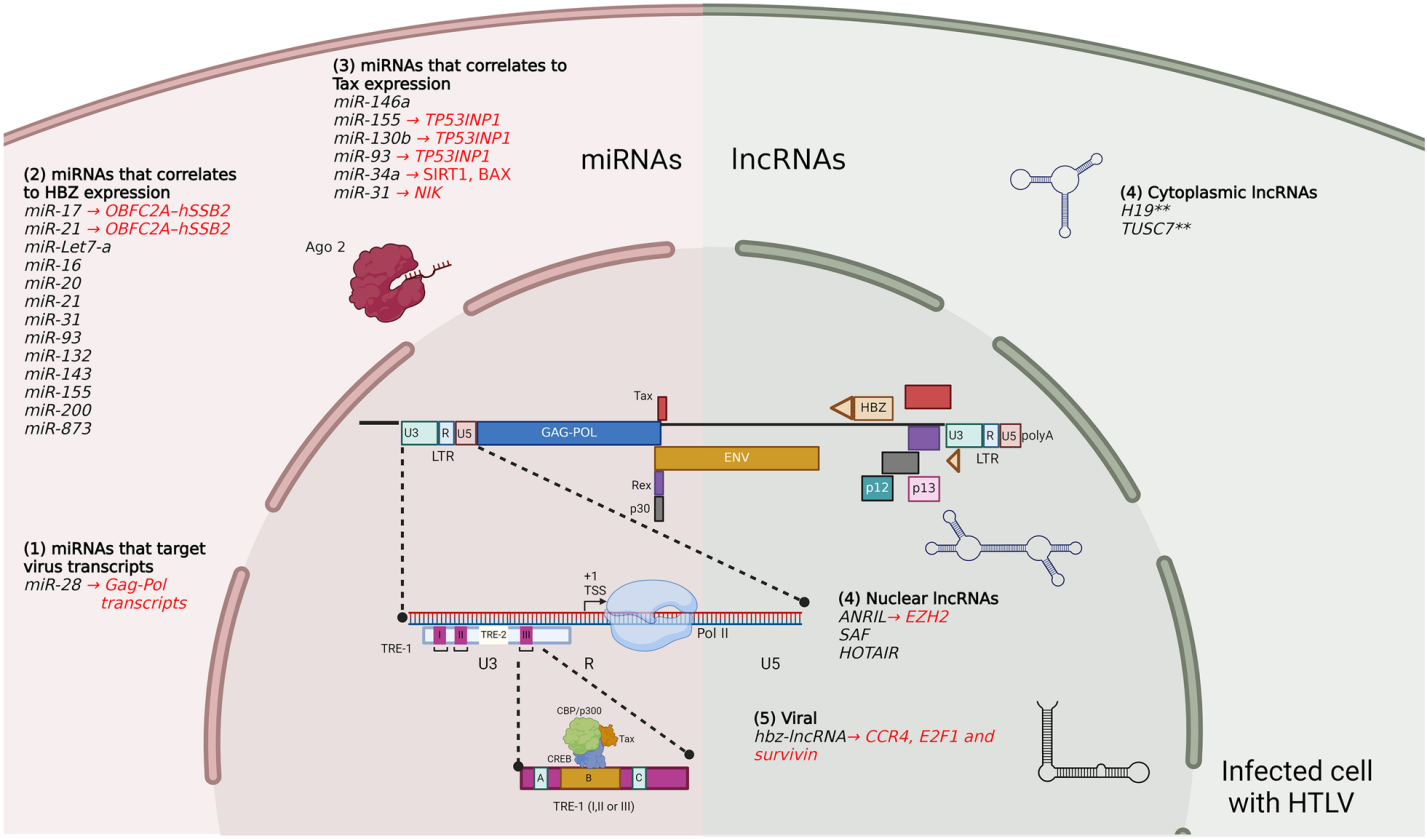
Dysregulated miRNAs and lncRNAs in HTLV infected cells. A schematic diagram of the HTLV genome within an infected cell highlighting the transcription processes along with the dysregulation of miRNAs and lncRNAs triggered by the virus infection. During the late phase of HTLV infection, Tax protein migrates to the cell nucleus and interacts with the Transcriptional Response Element (TRE) 1 through the Tax/CREB/CBP/p300 complex promoting the transactivation process. As a consequence of multiple virus transcription and replication rounds, distinct cellular miRNAs and lncRNAs are dysregulated or functionally-sequestered provoking multiple changes in the host cell. Importantly, because of the nature of the HBZ transcript, it also has lncRNA functions. Within the schematic, the different miRNAs and lncRNAs that are dysregulated or functionally sequestered are listed as follows. miRNAs that 1) target virus sequences 2) have a dysregulation that correlates to HBZ expression, 3) have a dysregulation that correlates to Tax expression, 4) dysregulated or functionally-sequestered (nuclear or cytoplasmic) lncRNAs, 5) viral HBZ-lncRNA. ** The subcellular location and interaction of these lncRNAs has not been specified. Figure created with Biorender

HTLV and lncRNAs constitute a relatively new field under investigation, where the available evidence is limited (Fig. 2; Table 2). HTLV-infected adult T cell leukemia lymphoma (ATLL) cells express the antisense HBZ but only a minor fraction is translated. Recent evidence revealed that a significant proportion of antisense HBZ transcripts are inefficiently polyadenylated, leading to their nuclear retention, reminiscent of mechanisms observed in nuclear retention of lncRNAs [162]. The HBZ transcripts in the nucleus then bind to promoters of CCR4, transcription factor E2F1 and survivin genes that contribute to viral persistence and stimulate proliferation of HTLV-infected cells [162, 163]. Host lncRNAs can also be affected by HTLV, for example differences between lncRNAs in HTLV-infected T-cell lines compared to uninfected T cells were observed in which host lncRNA antisense noncoding RNA in the INK4 locus (ANRIL), H19, and SAF presented heightened levels whereas HOTAIR and TUSC7 were slightly reduced [164]. Among these lncRNAs, ANRIL shows the highest expression and interacts with EZH2, a histone methyl transferase highly expressed in several neoplasms and cancers [165]. The interaction between EZH2, ANRIL along with ReIA/p65 factors in ATLL cells leads to persistent activation of the NF-κB pathway in a methyl transferase independent manner in the absence of viral Tax [164]. In addition, EZH2-ANRIL can inhibit apoptosis by suppressing p21/CDKN1A in a methyl transferase dependent manner by inhibiting apoptosis and increasing the survival phenotype [164]. Hence, both HBZ-like lncRNA and HTLV-induced cellular lncRNA ANRIL support cell proliferation and survival, potentially contributing to the malignant phenotype.

**Table 2:** Target and effects of cellular lncRNAs and miRNAs during HTLV transcriptional and post transcriptional events

lncRNA	Target	Outcome	References
hbz (viral)	CCR4, E2F1 and survivin	Promotes virus latency	[162, 163]
ANRIL	EZH2	Persistent activation of	[164]
		NF-κB pathway	

miRNA	Target	Outcome	References
miR-28	Gag-pol RNA	Downregulation of HTLV	[166]
miR-155	TP53INP1	Apoptosis control	[173, 175]
miR-130b, miR-93			
miR-34a	SIRT1, BAX	Apoptosis control	[176]
miR-31	NIK	Pro-apoptosis	[177]
miR-17, miR-21	OBFC2A–hSSB2	Abnormal cell proliferation	[181]

### Host miRNAs targeting HTLV RNA

There is evidence that cellular miRNAs could target HTLV genomic RNA. The first reported miRNA interfering with HTLV expression was miR-28-3p that targets the genomic gag/pol mRNA and is highly expressed in resting T cells [166]. miR-28-3p can repress the expression of HTLV in a reporter assay but much less that of HTLV subtype 1A ATK1 strains, which present a mutation in the miR-28-3p binding site. The restriction by miR-28 to HTLV transmission occurs by interfering with the process of reverse transcription [166]. Nevertheless, considering that HTLV Tax and HBZ are pivotal proteins for HTLV pathogenesis and the development of the malignant phenotype ATLL or HTLV-associated myelopathy/tropical spastic paraparesis (HAM/TSP) [167], more miRNAs contribute to HTLV-induced disease by targeting the pathways that enhance their expression.

### miRNAs as contributors to cell proliferation and oncogenicity correlated with Tax expression

The persistent activation of the NF-κB pathway by Tax drives the abnormal induction of cytokines/chemokines, regulators of apoptosis, adhesion molecules, cell cycle regulators and miRNAs, which promotes malignant cellular transformation [168, 169]. Distinct miRNAs have been reported to be positively regulated by NF-κB in cells expressing Tax, which results in persistent downregulation of targeted genes [169]. For instance, miR-146a concentration is increased several folds by Tax, promoting the overall proliferation of HTLV-infected T cells [170, 171]. This heightened level of miR-146a correlates with cell transformation in other cell types, leading to cancer in some cases [172]. Another example is miR-155, whose expression is increased in HTLV-expressing cells. miR-155 expression is enhanced by Tax through NF-κB and activator protein-1 (AP-1) binding to its promoter and it favours the proliferation of HTLV-infected cells [173]. miR-155 acts in part by targeting the tumor protein 53-induced nuclear protein 1 (TP53INP1), a cell protein that induces apoptosis [174]. This miRNA potentially synergizes with miR-130b and miR-93 that also target TP53INP1 and whose expression is also increased in HTLV-infected cells [175]. HTLV positive cell lines also have elevated levels of miR-34a through the activation of its promoter by NF-κB and p53. miR-34a targets SIRT1 and the pro-apoptotic factor BAX, likely contributing to cell proliferation of HTLV infected cells [176]. In contrast, the pro-apoptotic miR-31 that represses the NF-κB pathway by targeting NF-κB inducing kinase (NIK) is profoundly repressed in ATLL. Consequently, miR-31 is a tumor suppressor whose suppression contributes to oncogenic signaling and inflammation in ATLL [177]. Overall, the dysregulation of these miRNAs by HTLV favors its replication and cell survival.

During HTLV transcription, Tax binds to the complex CBP/p300 and P/CAF resulting in histone acetylation and chromatin remodeling favoring virus transcription [74, 178]. miR-149 and miR-873 are negative regulators of the chromatin remodeling enzymes CBP/p300 and P/CAF. In HTLV-transformed MT-2 cells, there is a significant downregulation of miR-149 and miR-873 compared to uninfected cells suggesting their contribution to HTLV transcription/replication [179]. The overexpression of miR-149 and miR-873 represses CBP/p300 and P/CAF with a concomitant decrease of virus titers in cell supernatants [179]. However, these results should be taken with caution until further confirmation as an expression of concern was published about the methodology and microarray data availability [180].

### miRNAs as contributors to cell proliferation and genomic instability correlated with HBZ expression

While Tax is highly expressed only during HTLV replication, HBZ levels remain constant during replication and latency [79]. Therefore, HBZ could be involved in maintaining cell proliferation and silencing virus expression. A differential miRNA expression profile between CD4^+^ lymphocytes from HAM/TSP and uninfected patients showed an increase of miR-17 and miR-21 expression in HAM/TSP individuals correlated with HBZ, but not Tax expression [181]. Downstream analyzes of these miRNAs unveiled their involvement in the negative regulation of the DNA-damage effector OBFC2A–hSSB2, which was associated with abnormal cell proliferation and genomic instability [181]. Following the observation that 59 out of the 61 analyzed miRNAs were downregulated in primary ATLL cells [177], the relationship to HBZ expression was examined. Indeed, HBZ downregulates Dicer transcription by removing JunD, an AP-1 transcription factor from the Dicer proximal promoter. Among the most highly downregulated in ATLL, miR-let-7a, miR-16, miR-20, miR-21, miR-31, miR-93, miR125a, miR-132, miR-143, miR-155, miR-200 and miR-873, HBZ had a direct involvement through the dysregulation of the Dicer promoter [182].

Other miRNAs that could be involved in replication, latency and cell transformation have been recently discovered by high-throughput analyzes of differentially expressed miRNAs. An in-depth miRNA meta-analysis conducted in silico using the GSE28626, GSE31629, and GSE11577 datasets demonstrated significant differential expression of miR-let-7a, miR-let-7g, miR-181b, miR-26b, and miR-30c between individuals with HTLV-ATLL and healthy donors. This analysis suggested that these specific miRNAs play a role in the context of HTLV infection and may potentially serve as important markers or targets for further study and potential therapeutic interventions [183]. Another recent in silico work reviewed 42 differentially expressed miRNAs previously reported in healthy, HTLV-infected and ATLL individuals and analyzed them [184]. The results showed that 12 miRNAs (miR-34a-5p, miR-146b-5p, miR-181b-5p, miR-26a-5p, miR-26b-5p, miR-222-3p, miR-155-5p, miR-193a-5p, miR-199a-3p, miR-199b-3p, miR-423-5p, miR-150-5p) may have a major impact in distinct biological pathways as defined by the Kyoto encyclopedia of genes and genomes (KEGG) such as hsa05200: cancer; hsa04310: WNT signaling; hsa04010: MAPK signaling; hsa04350: TGF-β signaling; hsa04014: Ras signaling, suggesting possible contributions to ATLL phenotype [184].

## ncRNAs in other retroviral infections

ncRNAs also have diverse roles in the expression of endogenous retroviruses (ERVs) and in other animal retroviral infections [22]. ERV-derived lncRNA positively regulates antiviral responses (lnc-EPAV) acts as a positive regulator of NF-κB, triggering innate immune responses upon Sendai virus (SeV) or Vesicular Stomatitis virus (VSV) infection [185]. Human endogenous retrovirus subfamily H (HERVH) generates nuclear lncRNAs required for maintenance and acquisition of pluripotency in human somatic cells [186]. Bovine leukemia virus (BLV) encodes a conserved miRNA, BLV-miR-B4 which may be associated with B-cell neoplasms, a common BLV-associated tumor in cattle [187]. Avian leukosis virus subgroup J (ALV-J) encodes E (XSR) miRNA whose possible roles are involved in ALV-J pathogenesis and neoplastic myeloid cell transformation [188]. In silico assays of miRNAs derived from Rous sarcoma virus (RSV) suggest that a cluster of miRNAs potentially targets 8 tumor suppressor factors favoring the formation of sarcoma in roosters [189]. Host lncRNAs and miRNAs can also be used or regulated by retroviruses. lncRNA 7SL is packaged in murine leukemia virus (MLV); although the role of 7SL recruited to virions has not yet been elucidated, this lncRNA could act as a scaffold for virus particle formation [190]. A study with a high number of infected mice with MLV revealed that provirus integration within a specific mir-17–92 cistron is associated with retrovirus-mediated induction of host oncogenic miRNAs [191].

## ncRNAs as potential therapeutic strategies against HIV and HTLV

Considering the large number of miRNAs and lncRNAs involved in retroviral expression or latency, strategies based on their properties can be established to use them or their synthetic counterparts as possible treatments against the viruses.

### ncRNAs to make HIV or HTLV resistant cells

Based on five reported cases of an HIV cure after allogeneic hematopoietic stem cells (HSC) transplant (HSCTs), efforts have been made to make HSCT cures accessible to all HIV infected individuals by modifying a person’s own cells to make them resistant to HIV in an autologous HSCT. This could be achieved by the permanent expression of antiviral genes [192]. Small non-coding anti-HIV RNAs comprise the most diverse set of anti-HIV gene therapy candidates. They include decoy RNAs that mimic the TAR and RRE structures in HIV RNA, RNA aptamers designed to target HIV enzymes, ribozymes (Rzs), which catalyze RNA cleavage, shRNAs, which recruit the RNAi machinery to cleave their target RNA and U1 interference RNAs (U1i RNAs), which inhibit polyadenylation or enhance mRNA splicing [192–199]. In the case of HTLV infections, autologous HSCT gave little success while allogeneic HSCT resulted in an average of 3-years relapse from ATLL [200, 201]. RNA therapy approach against HTLV is exemplified by siRNAs targeting NF-κB delivered by nanoparticles that were able to decrease the size of Tax-induced tumors in mice [202]. These strategies could be used to express antiviral miRNAs and lncRNAs transduced with lentiviral vectors for permanent expression in HIV or HTLV target cells.

### ncRNAs targeting HIV transcription as potential treatment

In addition to post transcriptional gene silencing (PTGS) using micro- si- and shRNAs, small ncRNAs can use the RNAi machinery for transcriptional gene silencing (TGS). In TGS, small ncRNAs such as short antisense (as)RNAs, shRNAs, siRNAs and miRNAs can direct Ago proteins to a DNA target for sequence specific silencing [203]. This leads to epigenetic modifications that can be passed on in subsequent rounds of cell replication and induce a strong long-lasting repression of gene expression, making TGS an ideal mechanism to permanently block the expression of retroviruses such as HIV and HTLV. Several ncRNAs have been designed to use TGS to silence HIV expression by targeting different sequences in the 5′LTR. siRNAs targeting the HIV 5′LTR promoter could provide a prolonged suppression of HIV replication in chronically infected cells [204]. Based on these siRNAs, shRNAs were also permanently expressed in cells after delivery with a retroviral vector and shown to strongly suppress HIV gene expression in T cells and induced pluripotent stem cell (iPSC)-derived macrophages [205, 206]. One of these shRNAs, called PromA, targeting NF-κB binding sites, was effective at inhibiting HIV replication in immunocompromised mice transplanted with human PBMCs transduced with a gene expressing the shRNA [207]. It was also effective at inhibiting HIV replication and preserving hematopoietic and CD4^+^ T cell populations when expressed in human cord-derived HSCs transplanted into immunocompromised mice [208]. A combination of siRNAs or shRNAs targeting the LTR promoter was also shown to provide a robust resistance to HIV reactivation by different stimuli suggesting that this mechanism could permanently lock the provirus in a latent state [209, 210].

Other target sites for siRNAs and shRNAs in the HIV promoter include LTR-247 and LTR-362, named based on their position within the reference HIV strain HXB2 [211, 212]. shRNA LTR-362, which overlaps with PromA, strongly inhibited HIV replication in PBMCs with no off-target effects [213, 214]. Additional shRNAs designed to specifically target HIV clade C variants were also effective at inhibiting HIV replication in PBMCs, showing that this strategy can be easily modified to target infections caused by diverse HIV strains [215]. Overall, the use of TGS inducers such as siRNAs and shRNAs could be utilized to cure HIV, and potentially HTLV, by locking the proviruses in a latent state. This mechanism, particularly when induced by shRNAs could also be used in combination with other small non-coding RNAs to generate HIV or HTLV resistant cells to cure the infections [216].

### Strategies using miRNAs and lncRNAs for locking retroviruses in a latent form

Based on the identified miRNAs that inhibit HIV expression and/or maintain latency in resting primary CD4^+^ T lymphocytes and macrophages, miRNAs (miR-28, 29a, 29b, 125b, 382, 150, 223, 155, 196b, 1290 and 642a) could be synthesized as miR mimics and tested for their long-term inhibition of HIV replication in lymphocytes. By doing so, their capacity to establish and maintain latency could be tested in a “block and lock” strategy. Indeed, overexpression of miR-28, 125b, 150, 223 and 382 directly inhibit HIV replication [136, 137]. Similarly, miR-29a has a strong ability to reduce HIV replication by targeting the Nef sequence and could be used as a pro-latency miR mimic [138, 139]. miR-29b, miR-149, miR-138 and miR-326 maintained a long-term decrease in HIV replication, while miR-326 targeting Nef-U3 region was much more effective when its sequence was optimized to reach full complementarity with HIV RNA [140]. miR-196b and miR-1290 mimics also effectively decreased HIV replication by targeting the Nef/U3 sequences [141]. miRNAs targeting transcription factors required for HIV could also be used to reduce viral replication. Indeed, miR-198, 27b, 29b, 150 and 223 decrease the expression of CycT1 [93, 142] whereas miR-642a reduces AFF4 mRNA and protein levels [143]. miR-15a, b, miR-16, miR-20a, miR-93, miR-106b mimics targeting Pur-α and miR-17, miR-20a targeting P/CAF could also directly decrease HIV transcription [141, 145, 146]. miR-155 targets several HDF, which could all contribute to the downregulation of HIV transcription [147, 148]. Assays of co-transfections of cells with miR-29a-3p, miR-155-5p, miR-642a-3p mimics and HIV NL4-3 decreased viral production suggesting that these miR mimics could be tested for long-term HIV inhibition in lymphocytes in a block and lock strategy. Another alternative would be to use anti-miRs to inhibit miRNAs that upregulate HIV transcription and replication (Table 1). For long-term inhibition, miRNA mimics targeting HIV RNA, transcription factors or HDFs, or anti-miRs against favourable HIV miRNAs could be expressed on a lentiviral vector and transduced in lymphocytes as with shRNAs. A strong and sustained inhibition has been observed with miR-29a, and optimised miR-326 targeting the Nef-U3 region and could be sequences of choice for latency induction [139, 140]. miR-28, 150, 223 and 382 targeting the virus and showing an increased expression in both CD4^+^ lymphocytes and monocytes could provide inhibition in a wider variety of cells [136, 137]. Furthermore, among miRNAs affecting cellular factors, miR-642a targeting AFF4 and miR-155 targeting different HIV HDFs (Table 1) show strong virus inhibition with no immediate effect on cell viability and could be excellent compounds for sustained HIV inhibition [143, 148].

So far, no clinical trials have been done to overexpress these miRNAs for a long period. In this regard, it is essential to determine the collateral effects, including the downregulation of targets and off-target effects by ncRNAs, along with optimizing their overexpression in preclinical and clinical trials to inform their potential use. For instance, miR-29a has been identified as a tumor-related miRNA whose overexpression is associated with the proliferation, invasion and metastasis of hepatocarcinoma and breast cancer cells [217, 218]. Conversely, low levels of miR-29a are correlated with T-cell acute lymphoblastic leukemia [219]. Another example is miR-642a that indirectly modulates transcription elongation by targeting AFF4 in the SEC, suggesting that this miRNA could be used to silence HIV transcription. However, disruption of any SEC component can cause aberrant interaction with mixed-lineage leukemia (MLL) protein, promoting the relocation of SEC to the MLL gene cluster and leading to aggressive acute leukemia [220]. Furthermore, CDK9, as part of pTEFb, contributes to various tumors by increasing the expression or hyperreactivity of different oncogenic factors [221]. In contrast, vmiRNAs targeting HIV might offer limited off-target effects, but they still need to be confirmed in biological assays and verified for innocuity.

In HTLV-infected cells, miR-28 that targets HTLV RNA or the pro-apoptotic miR-31 could be overexpressed to decrease viral replication or Tax-induced tumorigenicity, respectively [166, 177]. In contrast, anti-miRs that counteract the activity of miR-146a, miR-155, miR-130b, miR-93 and miR-34a on cell proliferation could be used to inhibit tumors induced by HTLV infection [171–175]. Similar to those targeting HIV, these miRNAs must undergo tests to determine the effective dose as well as their long-term tolerance.

lncRNAs that contribute to latency could also be overexpressed to block HIV expression. Overexpression of 7SK could sequester pTEFb and overexpression of NRON could co-opt NFAT as well as induce Tat degradation [111, 112]. Similarly, AK130181 can decrease HIV transcription by decreasing the availability of NF-κB [117]. Other lncRNAs acting indirectly on expression, like NEAT-1 which induces retention of HIV transcripts in the nucleus and GAS5 which acts as a sponge to inhibit miR-873, could also decrease viral replication [121, 122]. Blocking lncRNAs that have a positive effect on transcription or post-transcriptional regulation by antisense nucleotides, Rzs or shRNAs could be another therapeutic option to maintain latency. Inhibiting the activity of HEAL, MALAT-1 and uc002yug.2 would go in this direction [114, 115, 121]. Overexpressing or blocking the activity of lncRNAs will require a careful monitoring of their normal cellular function. Furthermore, although the lncRNA ANRIL contributes to cell proliferation and likely to the malignant phenotype of HTLV infection, the inhibition of its downstream targets EZH1/2 by small molecules is currently a preferred treatment for ATLL [222].

### Strategies using miRNAs and lncRNAs to reactivate HIV from latency to eliminate infected cells

Methods to reactivate HIV from latency followed by the destruction of reactivated cells are called the “shock and kill” strategy to eliminate the HIV-infected cellular reservoir. For this purpose, many chemical compounds can be used to reactivate latently infected cells [223]. For this strategy lncRNAs MALAT1, HEAL and uc002yug.2 have been proposed as LRAs for purging HIV reservoirs [224]. Indeed, MALAT1 and HEAL are transcriptional activators by relieving epigenetic repression mediated by histone methylation. MALAT1 is increased by LRAs and could mediate transcriptional reactivation [115]. MALAT1 also acts as a sponge to prevent miR-155 and miR150-5p inhibitory activity [116]. Overexpression of HEAL enhances HIV replication by transcriptional activation and could serve as an LRA [114]. lncRNA uc002yug.2 activates latent HIV through regulating alternative splicing of RUNX1 and increasing the expression of Tat. It was able to reactivate HIV from latently infected cells to a similar level as suberoylanilide hydroxamic acid or phorbol 12-myristate 13-acetate [121]. lncRNAs could also be used as targets and their silencing could reactivate HIV expression. In this line, PVT1 and RP11-347C18.3 have been suggested as a target for reactivation [123] and silencing AK130181 reactivated viral production from HIV latently infected Jurkat T cells and primary CD4^+^ T cells, suggesting that this silencing could induce the activation of latent HIV reservoirs [117]. Similarly, miRNAs that reactivate HIV transcription could be tested as LRAs.

### ncRNAs delivery strategies

As presented in this manuscript, various ncRNAs may serve as excellent candidates to control and/or eliminate HIV and HTLV in infected patients. Importantly, the success of delivering these molecules presents a major obstacle.

Depending on the chosen strategy, target cells for HIV and HTLV may need to be engineered to continuously express ncRNA or, in the case of reactivating latently infected HIV and HTLV cells they may need to be delivered systemically as drugs. As the main target cells for both HIV and HTLV are T lymphocytes, engineering these cells ex vivo to express antiviral ncRNAs could lead to a sustained therapy. Several clinical trials have been executed to evaluate long term ncRNA expression in CD4^+^ T cells for safety and efficacy in HIV infected individuals following ex vivo retroviral transduction of the ncRNAs with gammaretroviral vectors or lentiviral vectors [216, 225]. Gammaretroviral vectors are generally ineffective at transducing resting CD4^+^ T cells, which have a long intermitotic half-life [226] and have the potential to be oncogenic. 3rd generation self inactivating lentiviral vectors have become the vector of choice for T cell transduction and have so far been shown to be safe and effective in various clinical trials with several reaching the market for delivery of chimeric antigen receptors (CAR) to T cells for the treatment of hematological malignancies [227]. A limitation of lentiviral vectors pseudotyped with the vesicular stomatitis virus envelope G protein (VSV-G) is that they have low transduction efficiency in resting T cells. However, this can be overcome by using an alternative envelope protein such as that derived from HIV [228]. To provide a permanent life-long expression of antiviral ncRNAs, HSCs can be transduced with lentiviral vectors and several clinical trials have been conducted with combinations of ncRNAs delivered ex vivo in this manner during a HSC transplant [216, 225]. As HSCs give rise to all cells that can be potentially infected by HIV or HTLV, it is hoped that this kind of therapy could one day provide a functional cure or long-term remission for these viruses.

A lot less progress has been made in the systemic delivery of ncRNAs for the treatment of HIV or HTLV. However, with new technologies being developed for the delivery of RNA therapies for other indications a targeted delivery method could soon be available to delivery ncRNAs to all latently infected cells for potential reactivation strategies or to all infected cells for potential deep latency strategies. The study of extracellular vesicles has rapidly expanded, with exosomes now recognized for exhibiting differences in function and phenotype. Exosomes are increasingly acknowledged as biological targeting agents and drug delivery tools. Engineered actively cargo-loaded exosomes, with their ability to transmigrate tissue barriers, high yield capacity for intercellular cargo delivery, high biocompatibility, and low immunogenicity, might represent an excellent delivery molecule [229]. To target cells expressing HIV envelope protein, exosomes loaded with miRNAs are decorated on the outer membrane with single‐chain variable fragments (ScFv) of anti-HIV envelope (Env) [230]. The ScFv could be exchanged for CD4^+^ T cell resting markers to customize the exosome tropism. Also, synthetic lipid nanoparticles (LNPs), when combined with LRAs such as Tat, have been shown to be effective delivery systems. Further exploration of the use of LNPs would be ideal for the reactivation of HIV-1 reservoirs under ART [231]. Several other promising delivery methods for ncRNA therapeutics are currently under development [232].

## Conclusions

miRNAs and lncRNAs participate in the transcriptional and post-transcriptional events that contribute to the overall replication and pathogenesis of HIV and HTLV. In HIV-infected cells, a large number target the viral RNA, transcription factors, HDFs or HRFs and contribute to either an active replication cycle or latency. Their full characterization will contribute to finding strategies to either maintain the virus in a latent form or to reactivate it and eliminate infected cells. In HTLV-associated malignancies, miRNAs or lncRNAs are parts of the pathways that control cell proliferation and lead to either oncogenesis or apoptosis. Controlling these pathways will help find treatment options for ATLL or HAM/TSP.

## Data Availability

Not applicable.

## Abbreviations

HIV: Human immunodeficiency virus
HTLV: Human T cell leukemia virus
ncRNA: Non-coding RNA
miRNA: MicroRNA
lncRNA: Long non-coding RNA
RNAi: RNA interference
lincRNA: Long intergenic non-coding RNA
RNA Pol: RNA polymerase
NXF1: Nuclear export factor 1
TREX: Transcription export complex
dsRNA: Double-stranded RNA
siRNA: Small interfering RNA
shRNA: Short-hairpin RNA
pri-miRNA: Primary miRNA
DGCR8: DiGeorge syndrome critical region 8
pre-miRNA: Precursor miRNA
TRBP: TAR RNA binding protein
PACT: Protein kinase R activator
Ago: Argonaute
P bodies: Processing bodies
UTR: Untranslated region
LTR: Long terminal repeat
U3: Unique region 3
R: Repeated
U5: Unique region 5
TAR: Trans-activation response element
P-TEFb: Positive transcription elongation factor b
Cdk: Cyclin-dependent kinase
CycT1: Cyclin T1
LaRP7: La-related protein 7
MePCE: Methylphosphate capping enzyme
HEXIM: Hexamethylene bisacetamide-induced proteins
SEC: Super elongation complex
AFF: AF4/FMR2 Family Members
ENL: Eleven nineteen leukaemia
AF9: ALL1-fused gene from chromosome 9 protein
ELL2: Eleven nineteen lysine-rich leukaemia protein
RRE: Rev response element
CRM1: Chromosomal maintenance 1
DDX3: DEAD-box helicase 3
UPF1: Up frameshift protein 1
ORF: Open reading frame
NLS: Nuclear localization signal
TRE-1: Tax responsive element I
CRE: CAMP response element
CREB: CAMP response element binding protein
CBP: CREB-binding protein
P/CAF: P300/CBP-associated factor
TORC: Transducer of regulated CREB protein
RexRE: Rex-responsive element
HBZ: HTLV basic leucine zipper factor
ART: Antiretroviral therapy
MBD2: Methyl-CpG binding domain protein 2
UHRF1: Ubiquitin-like with PHD and RING finger domain 1
MLL: Mixed-lineage leukemia
NF-κB: Nuclear factor kappa B
NFAT: Nuclear factor of activated T cells
TCR: T cell receptor
PBMCs: Peripheral blood mononuclear cells
PP2A: Protein phosphatase 2A
ASP: HIV antisense protein
EZH2: Enhancer of Zeste homolog 2
DNTMT3α: DNA methyl transferase 3 alpha
HDAC: Histone deacetylase
NRON: Non-coding repressor of NFAT
HEAL: HIV-enhanced lncRNA
MDMs: Monocyte-derived macrophages
(MALAT-1: Metastasis-associated lung adenocarcinoma transcript 1
PRC2: Polycomb repressive complex 2
SOCS1: Suppressor of cytokine signaling 1
NEAT1: Nuclear enriched abundant transcript 1
RUNX1: Runt-related transcription factor 1
GAS5: Growth arrest-specific transcript 5
PVT1: Plasmacytoma variant translocation 1
vmiRNA: Viral miRNA
AATF: Apoptosis antagonizing transcription factor
HDF: Host dependency factor
HRF: Host restriction factor
TLR: Toll-like receptor
ADAM: A disintegrin and metalloproteinase
TNPO3: Transportin-3
LEDGF: Lens epithelium-derived growth factor
TRIM32: Tripartite motif containing 32
EP2: Enhancer binding protein 2
MeCP2: Methyl-CpG binding protein 2
TASK: TWIK-related acid-sensitive K
dNTP: Deoxyribonucleotide triphosphate
CCR: C–C chemokine receptor
SIRT1: Sirtuin 1
BLIMP-1: B lymphocyte-induced maturation protein-1
IL: Interleukin
EC: Elite controllers
TNF: Tumor necrosis factor
TRAF6: TNF receptor-associated factor 6
IRAK1: IL-1 receptor-associated kinase 1
MxB: Myxovirus resistance protein B
IFN: Interferon
IFTIM1: IFN induced transmembrane protein 1
ATLL: Adult T cell leukemia lymphoma
ANRIL: Antisense noncoding RNA in the INK4 locus
HAM/TSP: HTLV-associated myelopathy/tropical spastic paraparesis
AP-1: Activator protein-1
TP53INP1: Tumor protein 53-induced nuclear protein 1
NIK: NF-κB inducing kinase
KEGG: Kyoto encyclopedia of genes and genomes
ERV: Endogenous retrovirus
lnc-EPAV: ERV-derived lncRNA positively regulates antiviral responses
SeV: Sendai virus
VSV: Vesicular Stomatitis virus
HERVH: Human endogenous retrovirus subfamily H
ALV: Avian leukosis virus
RSV: Rous sarcoma virus
MLV: Murine leukemia virus
HSC: Hematopoietic stem cell
HSCT: HSC transplant
Rz: Ribozyme
U1i: U1 interference
PTGS: Post transcriptional gene silencing
TGS: Transcriptional gene silencing
iPSC: Induced pluripotent stem cell
LRA: Latency reversing agent
